# Ferroportin-Dependent Iron Homeostasis Protects against Oxidative Stress-Induced Nucleus Pulposus Cell Ferroptosis and Ameliorates Intervertebral Disc Degeneration *In Vivo*

**DOI:** 10.1155/2021/6670497

**Published:** 2021-02-10

**Authors:** Saideng Lu, Yu Song, Rongjin Luo, Shuai Li, Gaocai Li, Kun Wang, Zhiwei Liao, Bingjin Wang, Wencan Ke, Qian Xiang, Chao Chen, Xinghuo Wu, Yukun Zhang, Li Ling, Cao Yang

**Affiliations:** ^1^Department of Orthopaedics, Union Hospital, Tongji Medical College, Huazhong University of Science and Technology, Wuhan 430022, China; ^2^Department of Health Management Center, Union Hospital, Tongji Medical College, Huazhong University of Science and Technology, Wuhan 430022, China

## Abstract

Ferroptosis is a specialized form of regulated cell death that is charactered by iron-dependent lethal lipid peroxidation, a process associated with multiple diseases. However, its role in the pathogenesis of intervertebral disc degeneration (IVDD) is rarely investigated. This study is aimed at investigating the role of ferroptosis in oxidative stress- (OS-) induced nucleus pulposus cell (NPC) decline and the pathogenesis of IVDD and determine the underlying regulatory mechanisms. We used tert-butyl hydroperoxide (TBHP) to simulate OS conditions around human NPCs. Flow cytometry and transmission electron microscopy were used to identify ferroptosis, while iron assay kit, Perl's staining, and western blotting were performed to assay the intracellular iron levels. A ferroportin- (FPN-) lentivirus and FPN-siRNA were constructed and used to explore the relationship between FPN, intracellular iron homeostasis, and ferroptosis. Furthermore, hinokitiol, a bioactive compound known to specifically resist OS and restore FPN function, was evaluated for its therapeutic role in IVDD both *in vitro* and *in vivo*. The results indicated that intercellular iron overload plays an essential role in TBHP-induced ferroptosis of human NPCs. Mechanistically, FPN dysregulation is responsible for intercellular iron overload under OS. The increase in nuclear translocation of metal-regulatory transcription factor 1 (MTF1) restored the function of FPN, abolished the intercellular iron overload, and protected cells against ferroptosis. Additionally, hinokitiol enhanced the nuclear translocation of MTF1 by suppressing the JNK pathway and ameliorated the progression of IVDD *in vivo*. Taken together, our results demonstrate that ferroptosis and FPN dysfunction are involved in the NPC depletion and the pathogenesis of IVDD under OS. To the best of our knowledge, this is the first study to demonstrate the protective role of FPN in ferroptosis of NPCs, suggesting its potential used as a novel therapeutic target against IVDD.

## 1. Introduction

Intervertebral disc degeneration (IVDD) is a leading cause of low back pain [1], which approximately 80–90% of the global population suffer from, causing immense health and economic burdens [2]. The intervertebral disc (IVD) comprises an inner aggrecan-rich, gel-like nucleus pulposus (NP) and an outer collagen I-rich fibrocartilaginous annulus fibrosus, bordered superiorly and inferiorly by hyaline cartilaginous endplates [3]. Located in the inner disc, NP cells (NPCs) constitute the largest proportion of the cell types found in the NP and are responsible for the synthesis and secretion of the extracellular matrix (ECM) which, in turn, maintains the multiple biological functions of the spine. The depletion of NPCs and the subsequent degradation of the ECM are the primary etiology of IVDD [4]. Therefore, exploring the mechanisms underlying the depletion of NPCs is considered important. IVDD is a complicated process that involves tissue damage caused by age-related changes as well as multiple stress factors [5]. Studies have increasingly implicated oxidative stress (OS) in the initiation and progression of IVDD [5–7]. In addition, reactive oxygen species (ROS) are reportedly involved in the apoptosis, autophagy, and senescence of NPCs, which alter cellular phenotypes and contribute to disc degeneration [2, 8–11]. However, these mechanisms do not fully explain the decline of NPCs or the unsatisfactory performance of current IVDD treatment strategies.

The term ferroptosis, coined in 2012, describes a form of regulated cell death induced by the small molecule erastin, which inhibits the cystine-glutamate antiporter and causes the depletion of glutathione. Ferroptosis is characterized by iron-dependent excessive accumulation of lipid hydroperoxides, which is distinct from necrosis, apoptosis, and autophagy based on biochemical, morphological, and genetic criteria [12]. The main morphological characteristics seen under transmission electron microscopy (TEM) signifying ferroptosis include dense, smaller mitochondria with vestigial cristae and increased membrane density [12]. Ferroptosis could be effectively prevented via specific ferroptosis inhibitors as well as iron chelators, which blocks pathological cell death process in the kidney, cerebrum, and other organs [13]. Ferroptosis is involved in pathological cell death associated with ischemia-reperfusion injury, stroke, carcinogenesis, kidney injury, and degenerative diseases such as Parkinson's, Huntington's, and Alzheimer's diseases [14]. Additionally, some studies found that ferroptosis may also play an important role in IVDD [15, 16].

Ferroportin (FPN), a multitransmembrane protein, is the only known mammalian iron exporter that transports iron from the cytoplasm to the extracellular space [17]. FPN is highly expressed in hepatocytes, macrophages, and duodenal enterocytes, which are responsible for iron acquisition [17]. FPN plays an important role in maintaining cellular iron homeostasis and systemic iron homeostasis. Additional, altering the FPN expression could cause iron overload or iron deficiency [18]. Ward and Kaplan reported that knocking out FPN expression caused a reduction in the cycling of iron in red blood cell hemoglobin and reduced absorption of dietary iron, which resulted in iron deficiency [19]. Ma et al. demonstrated that intracellular iron accumulation in breast cancer cells, which involves a lack of FPN, contributes to ferroptosis via increasing lipid ROS production by way of Fenton's reaction [20]. In addition to being expressed in the liver, spleen, and duodenum, FPN is also found in the NP tissue of the human IVD in which its role remains undefined.

Metal-regulatory transcription factor 1 (MTF1) is responsible for regulating various metals at the cellular level, especially iron and zinc [21]. When required, MTF1 translocates from the cytosol into the nucleus and binds to the promoters of target genes to activate transcription [21]. Hinokitiol (4-isopropyl-tropolone) is a bioactive aromatic tropolone first isolated from the heart wood of *Chymacyparis taiwanensis*. As a component of essential oils, hinokitiol has been shown to resist OS and restore FPN function [22, 23], inhibit the activation of JNK, and increase the nuclear translocation of MTF1, which leads to an increase in copper-inducible metallothionein-I transcription [22, 24, 25].

In the current study, OS was induced in NPCs via exposure to tert-butyl hydroperoxide (TBHP). The results indicated that ferroptosis was involved in TBHP-induced human NPC death and IVDD. TBHP aggravated ferroptosis in NPCs by suppressing FPN expression via MTF1. Suppression of the JNK pathway using hinokitiol reversed the ferroptosis induced by the TBHP treatment. We explored the ferroptotic mechanism underlying OS-induced IVDD with the expectation of detecting potential treatment strategies for IVDD.

## 2. Materials and Methods

### 2.1. Isolation and Culture of Human NPCs

All experimental were approved by the Ethics Committee of Tongji Medical College, Huazhong University of Science and Technology (No. S214). Having obtained informed consent from all patients, normal NP tissues were acquired from 7 males and 6 females (*n* = 13), aged 11–24 years with a mean age of 15.8 years, who had undergone surgery for idiopathic scoliosis. Degenerative NP tissues were obtained from 6 males and 7 females (*n* = 13), aged 28–65 years with a mean age of 45.2 years, who underwent disc excision surgery for lumbar disc herniation. The degenerative grade of NP tissue samples was classified according to Pfirrmann grades using magnetic resonance images as previously described [7].

Human NPCs were isolated as described previously [26]. Briefly, NP tissues were sectioned into fragments and enzymatically digested with 0.25 mg/ml type II collagenase (Invitrogen) for 6 h. After being washed in PBS and centrifuged, the isolated NPCs were cultured in Dulbecco's modified Eagle medium (DMEM)/F12 (Gibco) containing 15% fetal bovine serum (Gibco) and 1% penicillin-streptomycin (Invitrogen). After identification using NPC markers (CD24, KRT18, Abcam), the second-passage cells were used for subsequent experiments. In our experiments *in vitro*, NPCs were exposed to 25, 50, and 100 *μ*M of TBHP (Sigma) for 6 h or 12 h. For the purpose of inhibiting ferroptosis, NPCs were treated with medium containing ferrostatin-1 5 *μ*M (HY-100579, MedChemExpress), liproxstatin-1 5 *μ*M (HY-12726, MedChemExpress), deferoxamine 100 *μ*M (HY-B0988, MedChemExpress), hinokitiol 8 *μ*M (HY-B2230, MedChemExpress), or SP600125 20 *μ*M, a specific inhibitor of JNK (HY-12041, MedChemExpress), and harvested for subsequent analysis.

### 2.2. Measurement of Cell Viability and Proliferation

Cell viability was performed using the Cell Counting Kit-8 (CCK-8) (C0037, Beyotime, Shanghai, China) according to the manufacturer's instructions. The absorbance was measured at 450 nm using a spectrophotometer (BioTek, Winooski, VT, USA). NPC proliferation was detected via the incorporation of EdU (5-ethynyl-2′-deoxyuridine) (C0078S; Beyotime, Shanghai, China) according to the manufacturer's instructions, and fluorescence images were acquired using a fluorescence microscope (Olympus, BX53, Melville, NY, USA).

### 2.3. Lipid ROS Analysis

Lipid ROS levels were assayed using C11-BODIPY 581/591 (Thermo Fisher, D3861) according to the manufacturer's instructions. Briefly, cells were treated with the intended compounds and 10 *μ*M C11-BODIPY 581/591, following which the preparation was incubated at 37°C for 30 min. Next, cells were washed thrice with PBS before being trypsinized and resuspended in fresh PBS. Fluorescence emission peaks were analyzed using a flow cytometer (BD Biosciences, San Jose, CA, USA). The peak from ~590 nm to ~510 nm is proportional to lipid ROS generation.

### 2.4. TEM

Cells were fixed in 2.5% glutaraldehyde (Sigma-Aldrich, USA) for 1 h, followed by fixation in 2% osmium tetroxide for 3 h. After washing, cells were stained with 0.5% uranyl acetate for 12 h. Following dehydration and polymerization, samples were cut into 70–90 nm ultrathin sections with an ultramicrotome (EM UC7, Leica) and observed using a transmission electron microscope (FEI, USA).

### 2.5. Measurement of Labile Iron Levels

Iron concentrations were measured using an iron assay kit (ab83366, Abcam, Cambridge, MA) following the manufacturer's instructions. After being treated with various compounds, cells were collected, homogenized in 5x volume of iron assay buffer on ice, and centrifuged (13,000 × *g*, 10 min) at 4°C. The supernatant was collected and 5 *μ*l of iron reducer was added to each sample and incubated for 30 min at 37°C. Next, 100 *μ*l of iron probe was added to each sample and incubated for 60 min at 37°C away from light. Absorbance was measured at 593 nm using a microplate reader (Thermo Fisher Scientific).

### 2.6. Perl's Prussian Blue Stain

The iron content in tissue sections was determined using an iron stain kit (ab150674; Cambridge, UK). Histological sections were deparaffinized and rehydrated. Next, the iron stain solution was produced by combining equal volumes of potassium ferrocyanide and hydrochloric acid solutions. Slides containing tissue sections were incubated in the solution for 3 min, washed with distilled water, stained using Abcam nuclear fast red solution for 5 min, and then washed with distilled water. Finally, the sections were dehydrated in 95% ethanol, followed by absolute ethanol. The final blue stain directly correlates with nonchelated iron in the human NP tissue.

### 2.7. Western Blotting Analysis

Total proteins, cytoplasmic proteins, and nuclear proteins were extracted from cultured cells using the corresponding kits (Beyotime, Shanghai, China) according to the manufacturer's instructions. The protein samples were incubated with the following primary antibodies: TFRC (1 : 500), FTL (1 : 500), DMT1 (1 : 1000), MTF1 (1 : 500), JNK (1 : 1000), beta-actin (1 : 5000), GAPDH (1 : 1000), and Histone 3 (1 : 1000) (all obtained from Proteintech), FPN (1 : 1000; Novus Biologicals), and Phospho-JNK (1 : 1000; Cell Signaling Technology). This was followed by incubation with a horseradish peroxidase- (HRP-) conjugated secondary antibody (1 : 2000; Abcam). Beta-actin, GAPDH, and Histone 3 were used for normalization.

### 2.8. Quantitative Reverse Transcription Polymerase Chain Reaction (RT-qPCR)

TRIzol reagent (Invitrogen) was used to extract total RNA from cultured cells according to the manufacturer's instructions. The following primers were used: Homo FPN (F: 5′-TCTTTGCTTGCGGTCCTGAT-3′, R: 5′-GAGCAAAACACCCAGCCATT-3′); Homo TFRC (F: 5′-AAATGCCCTCTCTGGTGACG-3′, R: 5′-AGCACGATCAGCACAAGTCT-3′); Homo DMT1 (F: 5′-AAAAGCGCAGACTGGATGGA-3′, R: 5′-CGATGGTAAGGGGAGGAGGC-3′); and Homo GAPDH (F: 5′-GGAGTCCACTGGCGTCTTCA-3′, R: 5′-GTCATGAGTCCTTCCACGATACC-3′). GAPDH was used for normalization.

### 2.9. RNA Interference

Knockdown of FPN in human NPCs was achieved via transfection of FPN-siRNA (siFPN) (RiboBio Co., Guangzhou, China) using Lipofectamine 2000 (Invitrogen) according to the manufacturer's instructions. After verifying high silencing efficiency, the NPCs were used as the treatment group.

### 2.10. Plasmids and Transfection

Overexpression of FPN or MTF1 in human NPCs was conducted via transfection of lentiviral plasmid generating Lenti-FPN and Lenti-MTF1, respectively, (GeneChem, Shanghai, China) according to the manufacturer's instructions.

### 2.11. Surgical Procedure

All procedures involving animal were conducted in accordance with the National Institutes of Health guidelines for the care and use of laboratory animals and were approved by the ethical standards of the Animal Experimentation Committee of Huazhong University of Science and Technology. A simple annulus fibrosus puncture rat model was developed as previously described [27, 28]. Briefly, 40 Sprague–Dawley rats (3 months old) were randomly divided into five groups (*n* = 8 for each group), which were obtained from the Laboratory Animal Center of Huazhong University of Science and Technology (Wuhan, China). After the rats were weighed and anesthetized with 2% (*w*/*v*) pentobarbital (40 mg/kg), the experimental level (Co7/8) was located by digital palpation on the coccygeal vertebrae and confirmed by counting the vertebrae from the sacral region in a trial radiograph. The tail skin was sterilized, and the tail vertebral disc of Co7/8 was punctured though the tail skin, parallel to the end plates with a 27-gauge sterile needle from the lateral side of the tail and was held with a 4 mm depth for 30 s. After surgery, DFO, Fer-1, or hinokitiol was diluted with normal saline and injected intraperitoneally for 8 weeks at a dose of 200 mg/kg/d, 150 mg/kg/d, or 150 mg/kg/d, respectively. The degeneration group was administered an equal amount of normal saline daily until the rats were killed. All animals were allowed free unrestricted weight bearing and activity.

### 2.12. Immunofluorescence Analysis

Immunofluorescence analysis was performed as previously described [29]. First, cells were fixed with 4% paraformaldehyde for 20 min, permeabilized in PBS containing 0.1% Triton X-100 for 3 min, saturated in PBS containing 3% bovine serum albumin for 1 h, and incubated with anti-MTF1 (1 : 50, Santa Cruz Biotechnology) overnight at 4°C. Subsequently, the cells were incubated with goat anti-mouse antibodies for 1 h. Nuclei were then costained with DAPI (4, 6-diamidino-2-phenylindole) for 5 min, and the results were visualized using an Olympus fluorescence microscope (Olympus, NY, USA).

### 2.13. Histological and Immunohistological Analyses in Human NP Tissues and Animal Models

NP tissue was fixed in 10% formaldehyde for 24 h, embedded in paraffin, and sliced into 4 *μ*m sections. For histological analysis, the sections were deparaffinized, rehydrated, and stained with hematoxylin and eosin (HE) and safranin-O (SO). Immunohistochemistry was conducted as previously described [30]. Sections were incubated with primary antibody (diluted 1 : 200) against FPN (NBP1-21502, Novus Biologicals), FTL (10727-1-AP, Proteintech), and MTF1 (25383-1-AP, Proteintech). Next, the sections were incubated with secondary antibody (Santa Cruz Biotechnology). For the negative control, the primary antibodies were replaced with buffer. Finally, three fields of each slide were randomly chosen for microscopic observation using microscopy (Olympus, Tokyo, Japan).

### 2.14. Statistical Analysis

Data are expressed as the mean ± SD of at least three independent experiments. Statistical analyses were performed using GraphPad Prism 7 software (La Jolla, CA, USA). Significance of difference was determined by unpaired *t*-test or one-way ANOVA. Significance was defined as *p* < 0.05.

## 3. Results

### 3.1. Ferroptosis Was Involved in TBHP-Induced Cell Viability Decline and ROS Accumulation in Human NPCs

**Figure 1:**
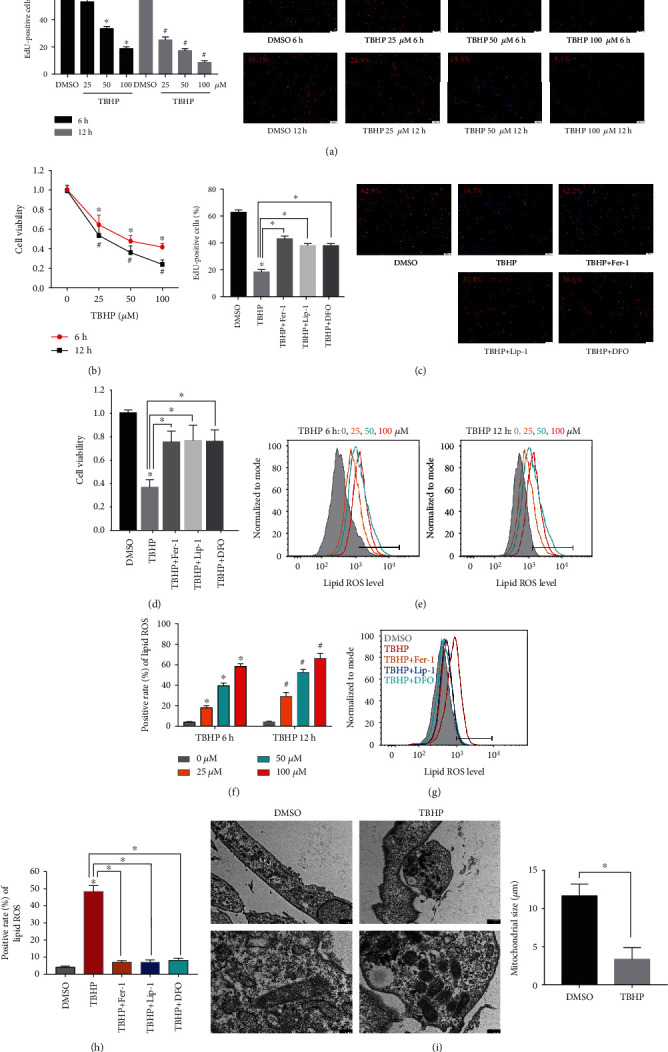
TBHP treatment induced cell viability decline, ROS accumulation, and ferroptosis in human NPCs. (a, b, e, f) The human NPCs were treated with TBHP (25–100 *μ*M) or DMSO for 6 h or 12 h. The quantitative values are expressed as mean ± SD (*n* = 3); ^∗^*p* < 0.05 versus DMSO (6 h), ^#^*p* < 0.05 versus DMSO (12 h). (a) Cell proliferation was detected using EdU staining under fluorescence microscope and the positive cells were quantitated. (b) Cell viability was examined by the absorbance of CCK-8. (c, d, g, h) The human NPCs were treated with DMSO, TBHP (50 *μ*M), Fer-1 (5 *μ*M), Lip-1 (5 *μ*M), or DFO (100 *μ*M) for 12 h. The quantitative values are expressed as mean ± SD (*n* = 3); ^∗^*p* < 0.05, TBHP versus DMSO; ^∗^*p* < 0.05, TBHP+ Fer-1/Lip-1/DFO versus TBHP. (c) Cell proliferation was detected using EdU staining under fluorescence microscope, and the positive cells were quantitated. (d) Cell viability was examined by the absorbance of CCK-8. (e–h) Lipid ROS levels were assayed using C11-BODIPY 581/591 by flow cytometry. (i) Observation of human NPC morphologic change after treatment with TBHP by TEM. The quantification of mitochondrial size is expressed as mean ± SD (*n* = 3); ^∗^*p* < 0.05 TBHP versus DMSO.

Previous studies have demonstrated that peroxide-induced cell death is involved in ferroptosis [31] and that the trigger of ferroptosis requires continuous formation of iron-dependent ROS over an extended period [12]. Therefore, we simulated OS conditions around human NPCs using various concentrations of TBHP for 6 h or 12 h. Cell viability was examined via EdU and CCK-8 assays to observe the impact of TBHP on human NPCs. As the TBHP concentration increased over a period of 6 h or 12 h, the viability of NPCs gradually decreased (Figures 1(a) and 1(b) ). However, TBHP-induced decline in the viability of NPCs was significantly rescued by the iron chelator deferoxamine (DFO) and ferroptosis inhibitors ferrostatin-1 (Fer-1) or liproxstatin-1 (Lip-1) [12] (Figures 1(c) and 1(d)). According to previous reports, lipid ROS accumulation is a hallmark of ferroptosis [12]. To verify this, lipid ROS were assessed using flow cytometry via the fluorescent probe, C11-BODIPY. The data indicated that treatment of NPCs with TBHP caused a dose-dependent and time-dependent increase in lipid ROS (Figures 1(e) and 1(f)). Similarly, lipid ROS accumulation was also suppressed by cotreatment with Fer-1, Lip-1, and DFO (Figures 1(g) and 1(h)). In addition, TEM observations indicated that TBHP-treated human NPCs contained smaller mitochondria with increased membrane density compared to the control group, a morphological characteristic of ferroptosis that is distinct from necrosis, apoptosis, and autophagy [12] (Figure 1(i)). These results suggested that ferroptosis was involved in TBHP-induced cell death in human NPCs, which was significantly alleviated by treatment with ferroptosis inhibitors and iron chelators.

### 3.2. Ferroptosis in Human NPCs Was Associated with FPN-Dependent Increase in Intracellular Iron Levels

**Figure 2:**
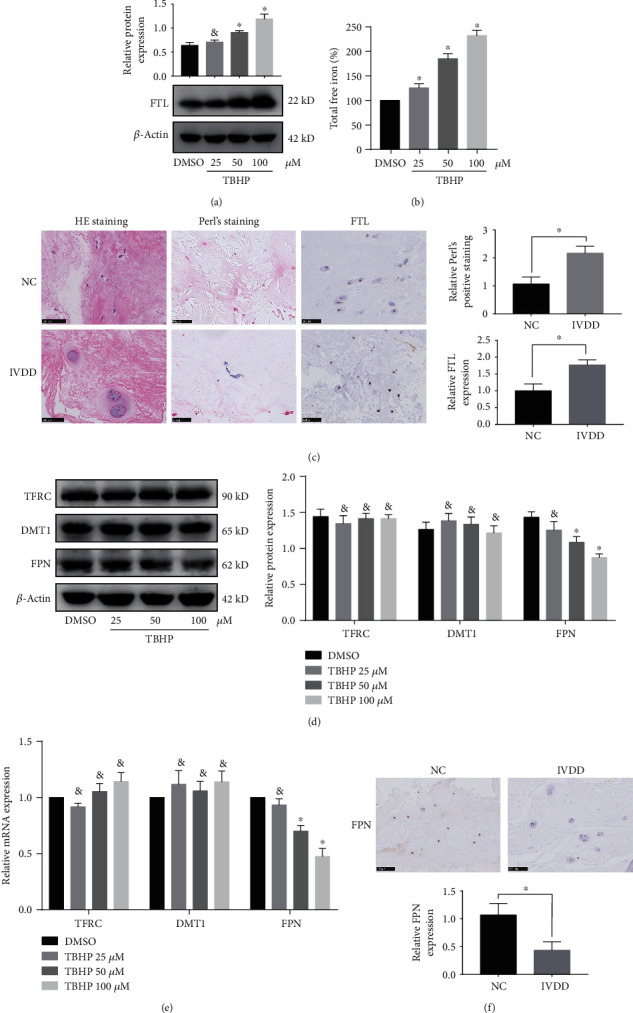
Ferroptosis in human NPCs was associated with intracellular iron overload and FPN dysfunction. (a, b, d, e) The human NPCs were treated with DMSO or TBHP (25–100 *μ*M) for 12 h. The quantitative values are expressed as mean ± SD (*n* = 3); ^∗^*p* < 0.05 versus DMSO. ^&^No significance versus DMSO. (a) FTL protein levels in human NPCs were determined using western blotting, which was normalized to *β*-actin. (b) Intracellular total free iron levels in human NPCs were assayed using iron assay kit. (c) Representative images of Perl's staining and FTL expression were performed in human NP tissues from the control (NC) group and IVDD group. Data are expressed as mean ± SD (*n* = 3); ^∗^*p* < 0.05, the IVDD group versus the control (NC) group. (d) TFRC, DMT1, and FPN protein levels in human NPCs were determined using western blotting, which was normalized to *β*-actin. (e) TFRC, DMT1, and FPN mRNA levels in human NPCs were determined by RT-qPCR, which was normalized to *β*-actin. (f) Representative images of FPN were performed in human NP tissues from the control (NC) group and IVDD group using immunohistochemistry analysis. Data are expressed as mean ± SD (*n* = 3); ^∗^*p* < 0.05, the IVDD group versus the control (NC) group.

Ferroptosis is an iron-dependent regulated cell death, and cellular iron is required for ferroptotic ROS accumulation [12]. To test the hypothesis that TBHP promotes ferroptotic ROS accumulation though an increase in intracellular iron levels, we determined the levels of labile iron pools (LIP) in human NPCs during exposure to different concentrations of TBHP. LIP can be measured indirectly by monitoring changes in cellular ferritin expression [32]. Western blotting results showed that TBHP treatment resulted in a significant increase in intracellular iron levels compared to that of the control, as indicated by the levels of ferritin light chain (FTL) in human NPCs (Figure 2(a)). To further verify the change in LIP, we directly measured LIP in human NPCs using an iron assay kit (Figure 2(b)), which showed that TBHP treatment led to a dose-dependent increase in intracellular LIP. Immunohistochemistry and Perl's staining of human NP tissues showed that iron in the IVDD group was increased compared to the control group (Figure 2(c)). These results suggested that intercellular iron overload plays an essential role in TBHP-induced ferroptosis of NPCs and IVDD.

Considering that the divalent metal transporter 1 (DMT1), transferrin receptor (TFRC), and FPN are mainly responsible for regulating the intercellular iron levels, we measured the corresponding mRNA and protein expression levels using RT-qPCR and western blotting, respectively. The results showed that FPN, but not TFRC or DMT1, was significantly downregulated in human NPCs treated with TBHP (Figures 2(d) and 2(e)), suggesting that it is FPN that mainly accounts for intercellular iron overload in human NPCs. In addition, we assessed the FPN level in human NP tissue specimens. Immunohistochemistry showed the expression of FPN decreased in the IVDD group compared to the control group (Figure 2(f)). These results demonstrated that FPN was downregulated in TBHP-induced human NPCs as well as human NP tissue of IVDD.

### 3.3. FPN Protected against TBHP-Induced Intercellular Iron Overload and Ferroptosis in Human NPCs

**Figure 3:**
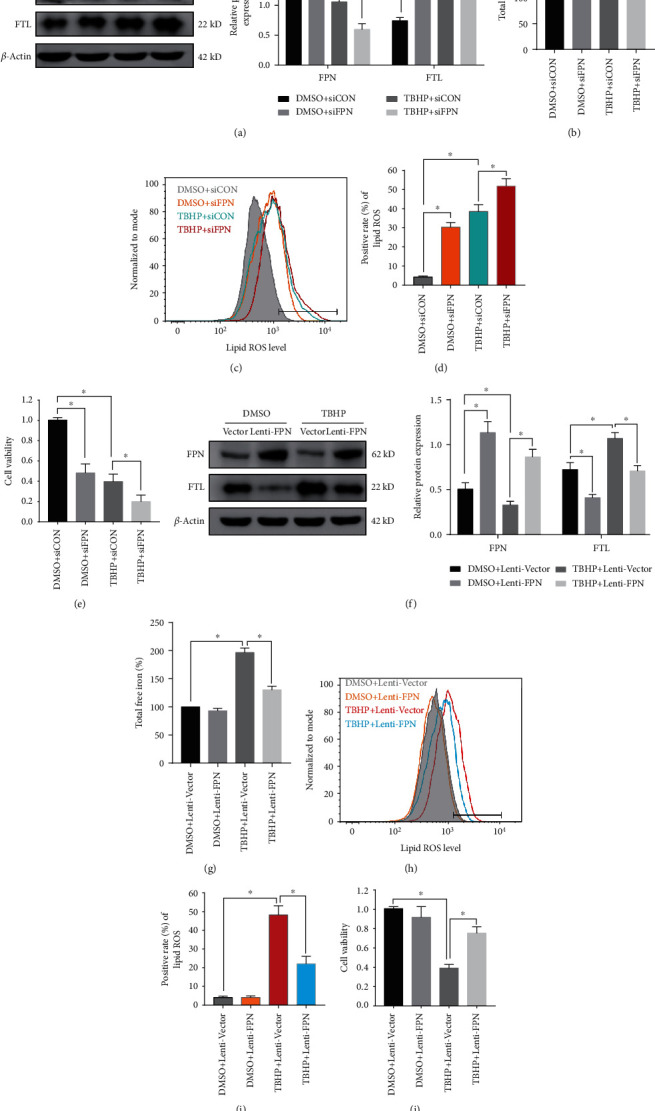
FPN protected human NPCs against intercellular iron overload and ferroptosis induced by TBHP treatment. (a–e) Scrambled siRNA (siCON) or FPN siRNA (siFPN) transfection was performed before DMSO or TBHP treatment (50 *μ*M) for 12 h. The quantitative values are expressed as mean ± SD (*n* = 3); ^∗^*p* < 0.05. (a) Representative western blotting assay and quantitation of the level of FPN and FTL protein, which was normalized to *β*-actin. (b) Intracellular total free iron levels in human NPCs were assayed using iron assay kit. (c, d) Lipid ROS levels were assayed using C11-BODIPY 581/591 using flow cytometry. (e) Cell viability was examined by the absorbance of CCK-8. (f–j) Lenti-Vector or Lenti-FPN infection was performed before DMSO or TBHP treatment (50 *μ*M) for 12 h. The quantitative values are expressed as mean ± SD (*n* = 3); ^∗^*p* < 0.05. (f) Representative western blotting assay and quantitation of the level of FPN and FTL protein, which was normalized to *β*-actin. (g) Intracellular total free iron levels in human NPCs were assayed using iron assay kit. (h, i) Lipid ROS levels were assayed using C11-BODIPY 581/591 by flow cytometry. (j) Cell viability was examined by the absorbance of CCK-8.

To further determine the role of FPN in generating intercellular iron overload and ferroptosis in human NPCs, we knocked down FPN using siFPN and overexpressed FPN by transfecting a lentiviral plasmid (Lenti-FPN) in control NPCs and TBHP-induced NPCs. Western blotting results showed the knockdown of FPN was efficient, and it increased intercellular iron levels in both control NPCs and TBHP-induced NPCs, indicated in ferritin and LIP (Figures 3(a) and 3(b)). FPN knockdown not only partially mimicked the effect of ferroptosis in control NPCs but also aggravated this effect in TBHP-induced NPCs (Figures 3(c)–3(e)). In addition, the results showed that Lenti-FPN infection significantly increased the expression of FPN and alleviated intercellular iron overload in TBHP-induced NPCs (Figures 3(f) and 3(g)). Lenti-FPN infection also alleviated ferroptosis in TBHP-induced NPCs (Figures 3(h)–3(j)). These data showed the intercellular iron overload and ferroptosis in TBHP-induced NPCs was associated with FPN dysfunction. FPN played a critical role in maintaining iron homeostasis and suppressing ferroptosis in human NPCs.

### 3.4. Suppression of MTF1 Downregulated FPN and Aggravated Ferroptosis in Human NPCs

**Figure 4:**
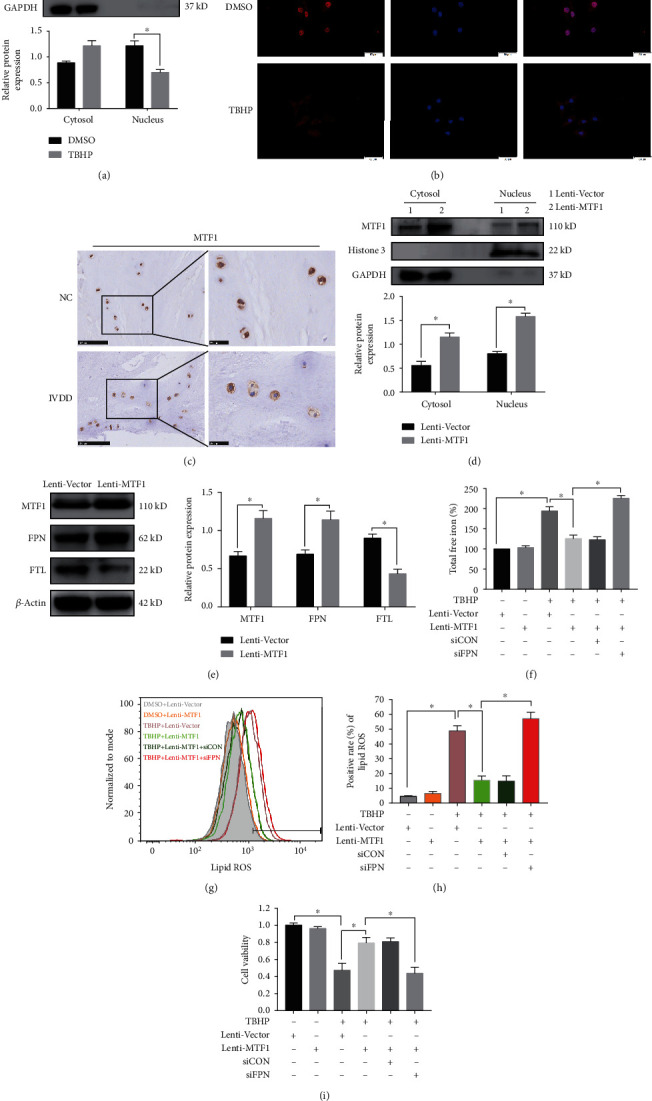
TBHP treatment aggravated FPN downregulation in human NPCs by MTF1 suppression. (a, b) The human NPCs were treated with DMSO or TBHP (50 *μ*M) for 12 h. The quantitative values are expressed as mean ± SD (*n* = 3); ^∗^*p* < 0.05. (a) MTF1 protein levels in cytosolic and nuclear fractions of human NPCs were determined using western blotting, which was, respectively, normalized to GADPH and Histone 3. (b) The human NPCs were fixed and stained with DAPI (nuclear) and MTF1 antibody. Representative images were shown to visualize the subcellular locations of MTF1. (c) The subcellular localization of MTF1 was performed in human NP tissues from the control (NC) groups and IVDD groups by immunohistochemistry analysis. (d, e) Lenti-Vector or Lenti-MTF1 infection was performed in human NPCs. The quantitative values are expressed as mean ± SD (*n* = 3); ^∗^*p* < 0.05. (d) MTF1 protein levels in cytosolic and nuclear fractions of human NPCs were determined using western blotting, which was, respectively, normalized to GADPH and Histone 3. (e) Representative western blotting assay and quantitation of the level of MTF1, FPN, and FTL proteins, which was normalized to *β*-actin. (f–i) Lenti-Vector or Lenti-MTF1 infection and scrambled siRNA (siCON) or FPN siRNA (siFPN) transfection were performed before DMSO or TBHP treatment (50 *μ*M) for 12 h. Data are expressed as mean ± SD (*n* = 3); ^∗^*p* < 0.05. (f) Intracellular total free iron levels in human NPCs were assayed using iron assay kit. (g, h) Lipid ROS levels were assayed using C11-BODIPY 581/591 using flow cytometry. (i) Cell viability was examined by the absorbance of CCK-8.

Next, we aimed to identify the regulator responsible for the decrease of FPN during TBHP treatment. Based on a literature search, MTF1 could regulate the genetic expression of FPN at the transcriptional level [21, 33, 34]. Western blotting results showed that TBHP treatment significantly reduced the nuclear fractions of MTF1 protein, but not of Histone 3 (nuclear loading control) (Figure 4(a)). Suppression of MTF1 nuclear translocation by TBHP treatment was further validated using an immunofluorescence assay (Figure 4(b)). In addition, immunohistochemistry showed decreased nuclear localization of MTF1 in the IVDD group compared to the control group (Figure 4(c)). To further confirm the role of MTF1 in the suppression of FPN, we overexpressed MTF1 by transfecting a lentiviral plasmid (Lenti-MTF1). Subsequent western blotting results indicated that Lenti-MTF1 infection upregulated MTF1 nuclear translocation, increased FPN protein levels, and decreased FTL protein levels and LIP in human NPCs (Figures 4(d)–4(f)). Consistently, overexpression of MTF1 also alleviated the ferroptosis phenotypes under TBHP treatment (Figures 4(f)–4(i)). To further confirm the essential role of FPN in MTF1 overexpression, we initiated FPN knockdown in Lenti-MTF1-infected NPCs. FPN knockdown significantly inhibited the protective effect of ferroptosis phenotypes in Lenti-MTF1-infected NPCs (Figures 4(f)–4(i)). These data indicated that TBHP treatment reduced the nuclear translocation of MTF1 in human NPCs, which resulted in the decreased expression of FPN and ferroptosis of NPCs.

### 3.5. Administering Hinokitiol Restored FPN Function and Suppressed TBHP-Induced Ferroptosis by Regulating the JNK/MTF1 Pathway

**Figure 5:**
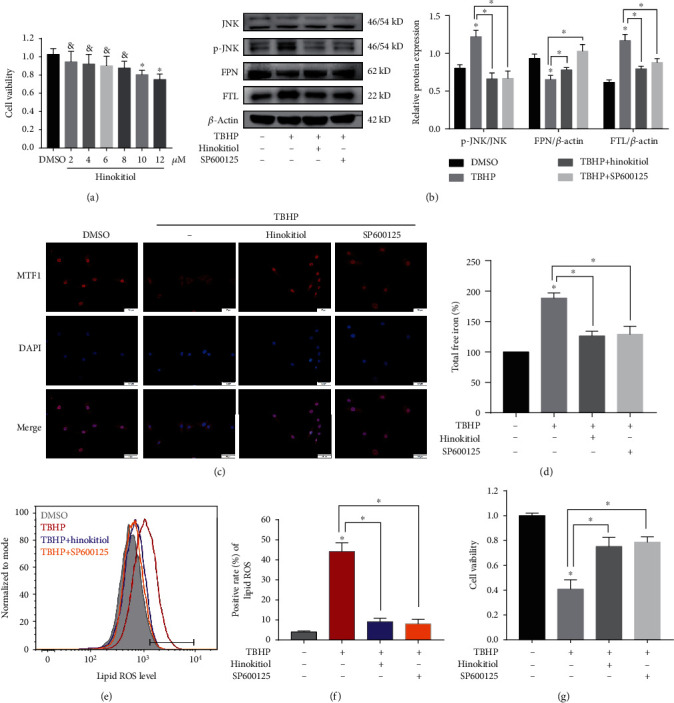
Hinokitiol restored FPN function and suppressed TBHP-induced ferroptosis by regulating the JNK/MTF1 pathway. (a) After treatment with various concentrations (2~12 *μ*M) of hinokitiol or DMSO, cell viability was examined by the absorbance of CCK-8 in human NPCs. The quantitative values are expressed as mean ± SD (*n* = 3); ^∗^*p* < 0.05 versus DMSO. ^&^No significance versus DMSO. (b–g) The human NPCs were treated with DMSO, TBHP (50 *μ*M), hinokitiol (8 *μ*M), or SP600125 (20 *μ*M) for 12 h. Data are expressed as mean ± SD (*n* = 3); ^∗^*p* < 0.05 TBHP versus DMSO, ^∗^*p* < 0.05 TBHP+hinokitiol/SP600125 versus TBHP. (b) Representative western blotting assay and quantitation of the level of p-JNK, FPN, and FTL proteins. P-JNK was normalized to JNK, while FPN and FTL were normalized to *β*-actin. (c) The human NPCs were fixed and stained with DAPI (nuclear) and MTF1 antibody. Representative images are shown to visualize the subcellular locations of MTF1. (d) Intracellular total free iron levels in human NPCs were assayed using iron assay kit. (e, f) Lipid ROS levels were assayed using C11-BODIPY 581/591 by flow cytometry. (g) Cell viability was examined by the absorbance of CCK-8.

A 12 h treatment with >10 *μ*M hinokitiol was cytotoxic to human NPCs, whereas doses with <8 *μ*M hinokitiol did not cause cytotoxicity (Figure 5(a)). Thus, we selected 8 *μ*M hinokitiol for the current study to determine its therapeutic role in protecting against ferroptosis of NPCs *in vitro*. Western blotting indicated that hinokitiol significantly suppressed activation of the JNK pathway by decreasing the phosphorylation level of JNK in a manner consistent with SP600125 (a specific inhibitor of JNK) (Figure 5(b)). We next investigated whether hinokitiol and SP600125 affected the nuclear translocation of MTF1 in human NPCs. MTF1 was found in both the cytosol and the nuclei of control NPCs using immunofluorescence assay (Figure 5(c)). Additionally, hinokitiol and SP600125 significantly rescued TBHP-induced suppression of MTF1 nuclear localization (Figure 5(c)). Furthermore, hinokitiol upregulated the expression of FPN in TBHP-treated NPCs, and SP600125 consistently exerted a protective effect (Figure 5(b)). Finally, hinokitiol and SP600125 also alleviated the ferroptosis phenotypes under TBHP treatment (Figures 5(d)–5(g)). Together, these data indicated that hinokitiol increased the nuclear translocation of MTF1 by suppressing the JNK pathway in TBHP-induced human NPCs, which resulted in an increase in FPN expression, a decline in intercellular iron levels, and the prevention of ferroptosis.

### 3.6. Suppressing Ferroptosis and Administrating Hinokitiol Ameliorated the Intervertebral Disc Degeneration *In Vivo*

**Figure 6:**
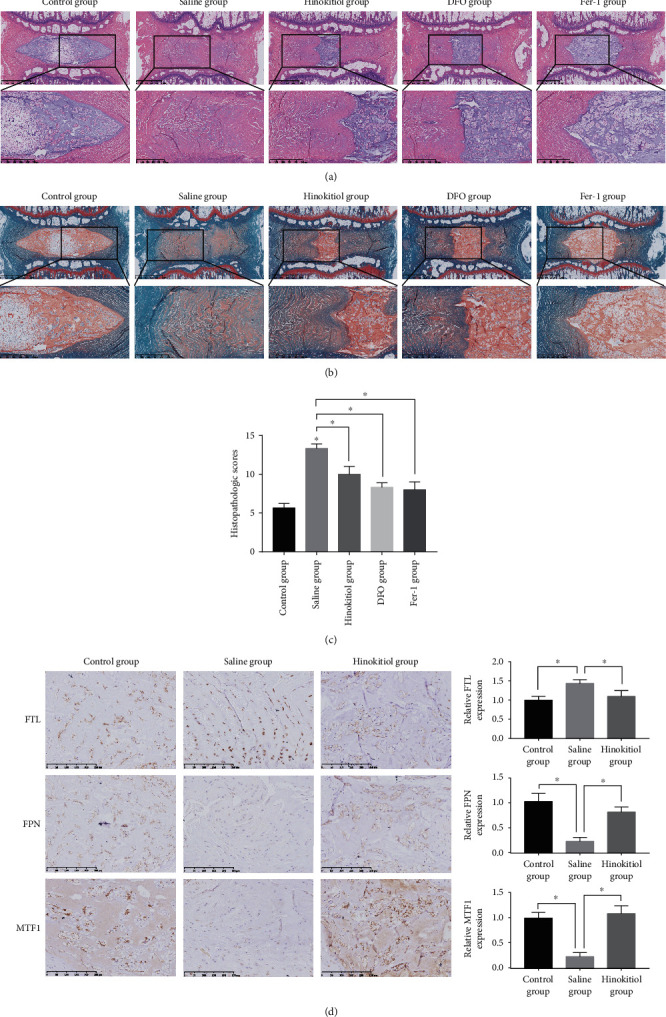
Ferroptosis occurred during IVDD, while administration of hinokitiol reduced the degree of disc degeneration *in vivo*. (a, b) Representative HE and SO staining images of the midsagittal sections of IVD specimens. (c) Histologic scores of the midsagittal sections of IVD specimens. Data are expressed as mean ± SD (*n* = 3); ^∗^*p* < 0.05, the saline group versus the control group; ^∗^*p* < 0.05, the DFO group/Fer-1 group/hinokitiol group versus the saline group. The interobserver error of each group is, respectively, 0.05 (control group), 0.02 (saline group), 0.05 (hinokitiol group), 0.03 (DFO group), and 0.07 (Fer-1 group). (d) Immunohistochemical staining for FTH, FPN, and MTF1 expressions in the rats' NP samples. Data are expressed as mean ± SD (*n* = 3); ^∗^*p* < 0.05, the saline group versus the control group; ^∗^*p* < 0.05, the hinokitiol group versus the saline group.

To further investigate the relationship between ferroptosis and IVDD and the therapeutic efficacy of hinokitiol *in vivo*, we constructed a tail disc percutaneous needle puncture animal model of IVDD using Sprague–Dawley rats as previously described [27, 28]. After 8 weeks of model operation, the midsagittal sections of IVD specimens from the animal models were subjected to histopathologic analysis and scoring. HE staining and SO staining showed that the structure of IVD degenerated in the saline group, DFO group, Fer-1 group, and hinokitiol group (Figures 6(a)–6(c)) and the degree of disc degeneration in the DFO group, Fer-1 group, and hinokitiol group was lesser than that in the saline group (Figures 6(a)–6(c)). Immunohistochemistry showed that FTL was increased in the saline group compared with the control group. Treatment with hinokitiol significantly alleviated the increase of FTL (Figure 6(d)). In addition, the nuclear translocation of MTF1 and the expression of FPN decreased in the saline group compared with the control group and were increased significantly when rats were treated with hinokitiol (Figure 6(d)). Taken together, these results revealed that ferroptosis and FPN dysfunction were involved in the pathogenesis of IVDD *in vivo*. Administration of hinokitiol may ameliorate IVDD progression though increasing the nuclear translocation of MTF1 and restoring the function of FPN *in vivo*.

## 4. Discussion

**Figure 7:**
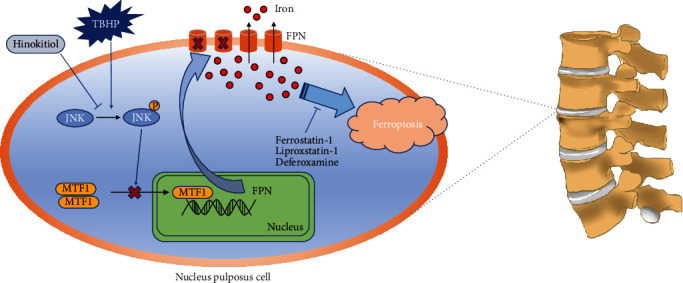
Schematic model illustrating the signaling pathway by JNK/MTF1/FPN to mediate TBHP-induced and hinokitiol-protective effect on human NPC ferroptosis.

Lower back pain has a negative impact on quality of life, with IVDD being its major cause. Although much effort has been made to diagnose lower back pain disorders in advance [35, 36], the available treatment is limited to muscle relaxants or nonsteroidal anti-inflammatory drugs to relieve symptoms [37, 38]. However, these drugs are unable to effectively alleviate IVDD or prevent its progression. The current study demonstrated the involvement of ferroptosis in the pathogenesis of IVDD. The decreased expression of FPN resulted in intercellular iron overload and ferroptosis in human NPCs (Figure 7). In addition, hinokitiol effectively suppressed ferroptosis and ameliorated IVDD via restoring the function of FPN both *in vitro* and *in vivo*.

OS is a common pathological phenomenon involved in various diseases, including diabetes, nonalcoholic fatty liver disease, and Alzheimer's disease [39]. The levels of ROS in the IVD increase significantly with the progression of IVDD. NPCs, the most important functional cells in the IVD, can utilize aerobic metabolism to produce ROS. OS mediated by excessive ROS accelerates the process of IVDD by multiple biological mechanisms [40]. He et al. studied that rat NPCs lose their ability to proliferate and become senescent, when exposed to sublethal hydroperoxide [40]. Yu et al. identified advanced glycation end products associated with IVDD that created OS and promoted mitochondrial dysfunction and apoptosis of NPCs [7]. Jiang et al. investigated autophagy and apoptosis as well as their interactions in NPCs under OS [9]. Other studies have determined that the expression of collagen type II and aggrecan was downregulated in hydroperoxide-induced NPCs [41]. However, these do not fully explain the decrease in NPCs and ECM degradation, as seen by the failure of drugs targeting these mechanisms to fully treat IVDD. Further research is needed to explore the mechanisms underlying IVDD.

Ferroptosis, an iron-dependent regulated cell death, was first identified while investigating cell death induced by the small molecule erastin, which inhibits the function of cystine-glutamate antiporter. Initial studies indicated that ferroptosis was biochemically, morphologically, and genetically distinct from necrosis, apoptosis, and autophagy [12]. However, recent studies have revealed that ferroptosis also is an autophagic process, which includes SQSTM1-mediated clockophagy, ROS-mediated autophagy, NCOA4-mediated ferroptinophagy, and degradation of GPX4 by chaperone-mediated autophagy [42–45]. Previous studies have reported that homocysteine promoted rat NPC death by ferroptosis, which was mediated by the upregulated methylation of GPX4 [15]. The current study used TBHP to simulate OS conditions. Lipid ROS, which are the hallmark of ferroptosis, significantly increased in TBHP-induced NPCs compared to that in noninduced cells. TEM showed that the distinctive morphological characteristics of ferroptosis were evident in TBHP-induced NPCs. TBHP-induced cell death was significantly declined when cells were coincubated with ferroptosis inhibitors and iron chelators. Additionally, ferroptosis inhibitors and iron chelators also ameliorated IVDD progression *in vivo*. This confirms the involvement of ferroptosis in TBHP-induced decline of NPCs and IVDD pathogenesis.

The sensitivity to ferroptosis is tightly linked to various biological processes, including lipid metabolism, iron metabolism, amino acid and glutathione metabolism, and the biosynthesis of GPX4, p53, coenzyme Q10, ferroptosis-suppressor-protein 1 (FSP1), and NADPH [14, 46–48]. Iron, a constituent of iron-sulfur proteins and hemoproteins, is required for the accumulation of lipid peroxides in the development of ferroptosis [49]. The export, import, storage, and turnover of iron impact ferroptosis sensitivity [14]. FPN is responsible for iron homeostasis at both systemic levels and cellular levels [50]. A previous study has reported that siramesine in combination with lapatinib induced ferroptosis via increased transferrin expression and decreased FPN expression, resulting in intercellular iron overload [20]. However, the role of FPN in human NPCs remains to be determined. The current study found that the expression level of FPN was reduced in TBHP-induced NPCs, degenerative human NP tissues, and degenerative rats' NP samples. Importantly, we further conformed that the reduced expression of FPN resulted in intracellular iron overload, which contributed to the development of ferroptosis.

FPN plays a critical role in iron homeostasis, and alterations in FPN may result in either iron overload or deficiency. The regulation of FPN expression is complex, with important layers of control at the transcriptional level via the regulation of levels and splice variants of mRNA, at the posttranscriptional level via an iron-regulatory element in the 5′-untranslated region of FPN mRNA and at the posttranslational level via direct interaction between FPN and hepcidin, a peptide hormone. These different regulatory mechanisms could influence FPN activity at its various sites of expression [19]. Previous studies have demonstrated that, at the transcriptional level, MTF1 can induce FPN transcription by translocating into the nucleus and binding to the FPN1 promoter in response to stress situations such as OS and heavy metal loading [33, 34]. We demonstrated that the nuclear translocation of MTF1 decreased in TBHP-induced NPCs, degenerative human NP tissues, and degenerative rats' NP samples. TBHP suppressed the nuclear translocation of MTF1, which resulted in the reduced expression of FPN, intracellular iron overload, and ferroptosis of NPCs.

Hinokitiol, a natural tropolone derivative, exhibits multiple biological activities such as antibacterial, antifungal, antiviral, anti-inflammatory, and anticancer. This compound has been used to treat decubitus ulcers, pulmonary tuberculosis, and lung gangrene in clinical practice [22, 51]. Hinokitiol inhibits platelet activation by suppressing the formation of hydroxyl radicals and attenuating the activation of Akt and MAPKs during antithrombotic activity *in vivo* [52]. Inhibition of JNK1/2 phosphorylation by hinokitiol suppressed mouse melanoma cell migration [22]. The current study demonstrated that hinokitiol increases nuclear translocation of MTF1, restores the function of FPN, and alleviates TBHP-induced NPC ferroptosis by suppressing the JNK pathway (Figure 7). Also, hinokitiol ameliorated IVDD progression *in vivo*. Our study has some limitations. Although our study found that TBHP attenuated ferroptosis in human NPCs by reducing FPN via MTF1, further investigation is warranted to elucidate the mechanisms underlying TBHP and MTF1 interaction. Furthermore, only a ferroptosis-associated decrease in human NPCs subjected to OS was investigated in our study. Additional investigations are warranted to confirm the universality of ferroptosis in IVDD via the simulation of other stress factors, such as inflammation, hypoxia, and compression.

## 5. Conclusions

To the best of our knowledge, the current study is the first to demonstrate the protective role of FPN in ferroptosis of human NPCs and the pathogenesis of IVDD. Furthermore, we revealed that TBHP downregulated FPN expression by reducing the nuclear translocation of MTF1, which causes intracellular iron overload and ferroptosis in human NPCs. Moreover, hinokitiol alleviated TBHP-induced OS and ferroptosis of NPCs by restoring the FPN function. Therefore, we inferred that repairing FPN function and inhibiting ferroptosis in human NPCs may be potentially effective therapeutic targets against OS-associated IVDD.

## Data Availability

The data used to support the findings of this study are included within the article.

## Abbreviations

CCK-8: Cell counting kit-8
DFO: Deferoxamine
DMT1: Divalent metal transporter 1
ECM: Extracellular matrix
Fer-1: Ferrostatin-1
FPN: Ferroportin
FSP1: Ferroptosis-suppressor-protein 1
FTL: Ferritin light chain
GAPDH: Glyceraldehyde-3-phosphate dehydrogenase
IVD: Intervertebral disc
IVDD: Intervertebral disc degeneration
JNK: c-Jun N-terminal kinase 1
Lip-1: Liproxstatin-1
LIP: Labile iron pools
MTF1: Metal-regulatory transcription factor 1
NP: Nucleus pulposus
NPCs: Nucleus pulposus cells
OS: Oxidative stress
ROS: Reactive oxygen species
TBHP: tert-Butyl hydroperoxide
TEM: Transmission electron microscopy
TFRC: Transferrin receptor
RT-qPCR: Quantitative reverse transcription polymerase chain reaction.
